# Superior laryngeal nerve block

**DOI:** 10.1016/j.bjorl.2026.101877

**Published:** 2026-07-22

**Authors:** Gustavo Polacow Korn, C. Blake Simpson

**Affiliations:** a Hospital Einstein Israelita, São Paulo, SP, Brazil; b University of Alabama-Birmingham (UAB), Birmingham, United States of America

The irritable larynx syndrome, first described in 1999, encompasses a spectrum of clinical conditions including chronic cough, muscle tension dysphonia, globus sensation, vocal fold dysfunction (also referred to as paradoxical vocal fold motion), laryngospasm, and throat clearing. This syndrome is characterized by alterations within the central nervous system that lead to a state of hyperexcitability in the sensorimotor pathways.^1^

The management of chronic cough, particularly neurogenic cough, a potentially debilitating condition, includes neuromodulatory medications ‒ often associated with potential side effects ‒ speech-language therapy, and Superior Laryngeal Nerve (SLN) block. Neurogenic cough is believed to result, at least in part, from a sensory neuropathy affecting the internal branch of the superior laryngeal nerve. Since the study by Simpson et al.^2^ in 2018, numerous subsequent studies have demonstrated the efficacy of this treatment in both the short and long term.

One of the main advantages of this technique is its generally safe application. The medications used are widely available, the procedure is quick, does not require specialized equipment, causes minimal patient discomfort, and, to date, no significant complications have been reported.

We suggest using a 27-gauge needle, and in the syringe a mixture of a long-acting particulate corticosteroid with a local anesthetic. The corticosteroid commonly used is triamcinolone (40 mg/1 mL) delivered as 1 ml, combined with 1 mL of either 1% lidocaine or 0.5% bupivacaine. The injection is performed at the posterior portion of the Thyrohyoid (TH) membrane near the exit of the SLN. There is some resistance at the TH membrane, which can often be perceived as the needle reaches the target nerve. This is the correct depth to deliver the block. The procedure is generally performed unilaterally, based on the patient’s perception of which side the irritation/discomfort is perceived in the neck prior to cough, although some authors advocate bilateral injections. The number of applications depends on the patient’s clinical response and evolution, but the authors urge caution with the use of a corticosteroid more than four times per year. For those patients receiving more than 4 injections per year, strong consideration should be given to lower the steroid dosage by either using a more dilute steroid (Trimacenolone 10 mg/mL) or leaving out the steroid altogether. Many patients on long-term nerve blocks do well with anesthetic-only preparations.

Muscle tension dysphonia represents another condition in which this block may be an excellent therapeutic option, particularly in patients who are refractory to conventional voice therapy.^3^ The procedure may also benefit patients presenting with paralaryngeal pain or odynophonia.^4^

In our experience, performing the injection in patients with muscle tension dysphonia may be more challenging due to increased laryngeal tension and a reduced thyrohyoid space. In such cases, manual manipulation to lower the larynx and increase this space may facilitate the procedure.

In patients with paradoxical vocal fold motion or exercise-induced laryngeal obstruction, the superior laryngeal nerve block may also represent a promising therapeutic option, although further studies are required to confirm its efficacy in these conditions.

Unlike the well-established use of the superior laryngeal nerve block in chronic cough, its application in other laryngeal disorders still requires additional investigation to better define its therapeutic role and clinical effectiveness.^5^

## ORCID ID

Gustavo Polacow Korn: 0000-0003-3718-204X

C. Blake Simpson: 0000-0002-0820-6201

## Funding

None.

## Conflicts of interest

The authors declare no conflicts of interest.

## Data availability statement

The authors declare that all data are available in repository.

## References

[1] Morrison M., Rammage L., Emami A.J. (1999). The irritable larynx syndrome. J Voice..

[2] Simpson C.B., Tibbetts K.M., Loochtan M.J., Dominguez L.M. (2018). Treatment of chronic neurogenic cough with in-office superior laryngeal nerve block. Laryngoscope..

[3] Benninger M.S., Quinton B.A., Tierney W.S. (2024). The application of superior laryngeal nerve block for non-cough laryngeal hypersensitivity. Laryngoscope..

[4] Tibbetts K.M., Dion G.R., Dominguez L.M., Loochtan M.J., Simpson C.B. (2022). In-office superior laryngeal nerve block for paralaryngeal pain and odynophonia. Laryngoscope..

[5] Peachman A.T., Root Z.T., Alsavaf M.B., Kim B., deSilva B.W., Matrka L. (2026). Prospective study of long-term outcomes and the patient experience with superior laryngeal nerve block for chronic cough. Laryngoscope.

